# The role of PD-1/PD-L1 and application of immune-checkpoint inhibitors in human cancers

**DOI:** 10.3389/fimmu.2022.964442

**Published:** 2022-09-13

**Authors:** Qing Tang, Yun Chen, Xiaojuan Li, Shunqin Long, Yao Shi, Yaya Yu, Wanyin Wu, Ling Han, Sumei Wang

**Affiliations:** ^1^Guangdong-Hong Kong-Macau Joint Lab on Chinese Medicine and Immune Disease Research, Clinical and Basic Research Team of Traditional Chinese Medicine (TCM) Prevention and Treatment of Non small cell lung cancer (NSCLC), Department of Oncology, The Second Clinical College of Guangzhou University of Chinese Medicine, Guangdong Provincial Hospital of Chinese Medicine, Guangdong Provincial Key Laboratory of Clinical Research on Traditional Chinese Medicine Syndrome, Guangzhou University of Chinese Medicine, Guangzhou, China; ^2^Department of Organ Transplantation, Second Affiliated Hospital of Guangzhou Medical University, Guangzhou, China; ^3^Institute of Rehabilitation Medicine, Shanghai University of Traditional Chinese Medicine, Shanghai, China; ^4^Department of Cerebrovascular Disease, Guangdong Provincial Hospital of Chinese Medicine, The Second Clinical College of Guangzhou University of Chinese Medicine, Guangzhou, China; ^5^Department of Oncology, The Second Affiliated Hospital of Guangzhou University of Chinese Medicine, Guangdong Provincial Hospital of Chinese Medicine, Guangzhou, China; ^6^State Key Laboratory of Dampness Syndrome of Chinese Medicine, The Second Affiliated Hospital of Guangzhou University of Chinese Medicine, Guangdong Provincial Hospital of Chinese Medicine, Guangzhou, China; ^7^Guangdong Provincial Key Laboratory of Clinical Research on Traditional Chinese Medicine Syndrome, The Second Affiliated Hospital of Guangzhou University of Chinese Medicine, Guangzhou, China

**Keywords:** PD-1/PD-L1, immunecheckpoint inhibitor, clinical application, biomarker, human cancers

## Abstract

Programmed cell death protein-1 (PD-1) is a checkpoint receptor expressed on the surface of various immune cells. PD-L1, the natural receptor for PD-1, is mainly expressed in tumor cells. Studies have indicated that PD-1 and PD-L1 are closely associated with the progression of human cancers and are promising biomarkers for cancer therapy. Moreover, the interaction of PD-1 and PD-L1 is one of the important mechanism by which human tumors generate immune escape. This article provides a review on the role of PD-L1/PD-1, mechanisms of immune response and resistance, as well as immune-related adverse events in the treatment of anti-PD-1/PD-L1 immunotherapy in human cancers. Moreover, we summarized a large number of clinical trials to successfully reveal that PD-1/PD-L1 Immune-checkpoint inhibitors have manifested promising therapeutic effects, which have been evaluated from different perspectives, including overall survival, objective effective rate and medium progression-free survival. Finally, we pointed out the current problems faced by PD-1/PD-L1 Immune-checkpoint inhibitors and its future prospects. Although PD-1/PD-L1 immune checkpoint inhibitors have been widely used in the treatment of human cancers, tough challenges still remain. Combination therapy and predictive models based on integrated biomarker determination theory may be the future directions for the application of PD-1/PD-L1 Immune-checkpoint inhibitors in treating human cancers.

## Introduction

PD-1 is a representative immunosuppressive checkpoint and mainly expressed in macrophages, B lymphocytes, dendritic cells (DCs), monocytes, tumor-specific activated T cells, myeloid cells and natural killer (NK) cells under conditions of chronic antigen exposure (1–3). PD-L1 is one of the PD-1 ligands. PD-L1 expression has been shown to be a valuable biomarker for the prognosis and prediction of the sensitivity of PD-1/PD-L1 inhibitors. The expression of PD-L1 is mainly expressed in tumor cells, tumor-infiltrating cells and antigen-presenting cells (APCs) in many cancers (1, 4). In recent years, a number of studies have confirmed the clinical significance of PD-1/PD-L1 antibodies and their prognostic impact on human cancers (5, 6). However, the relationship between this biomarker and its clinical significance is imperfect and varies in different types of human cancers (7).

In general, PD-1/PD-L1 inhibitory checkpoints suppress T cell receptor-mediated cytotoxicity and CD8^+^ T cell proliferation by interacting with the ligand PD-L1, thus avoiding the killing effect of the autoimmune system on tumor cells and immune surveillance (8–10). Immune checkpoint antibodies as promising cancer therapeutic strategies are based on their natural role acting as T cell-activated co-inhibitory receptors. Undoubtedly, the co-stimulatory and co-inhibitory receptors of T cells play an important role in the treatment of PD-1/PD-L1 immune checkpoint inhibitors (11). Expression of PD-L1 in tumor cells or tumor-associated stromal cells is a potential predictive marker for response and outcome of anti-PD-1/PD-L1 immunotherapy (1, 12).

Despite the remarkable efficacy of PD-1/PD-L1 immunocheckpoint inhibitors in the treatment of tumors, some problems also remain, such as drug resistance and adverse events. The presence of drug resistance significantly reduces the efficacy of anti-PD-1/PD-L1 immunotherapy. Exploring the mechanisms of PD-1/PD-L1 immunocheckpoint inhibitors resistance will contribute to the discovery of new immunotherapeutic strategies to control disease progression and provide a more sustainable survival benefit for patients (13). In addition, PD-1/PD-L1 immunocheckpoint inhibitors acting as immunomodulatory drugs can significantly enhance the natural defense of the immune system against cancers, while inevitably leading to some immune-related adverse events, the erroneous stimulation of the immune system leads to immune injuries to the normal tissues of the body (14). Therefore, in order to further improve the treatment outcome and reduce the risk of patients, it is necessary to learn more about the immune-related adverse events of PD-1/PD-L1 immunocheckpoint inhibitors in the treatment of human cancers.

In any case, we believe that tumor immunotherapy based on PD-1/PD-L1 inhibitors will become a promising strategy for human cancers. This article will focus on the role of PD-1/PD-L1 and the application of PD-1/PD-L1 inhibitors in human cancers.

## Mechanisms of tumor immunotherapy based on PD-1/PD-L1

**Figure 1:**
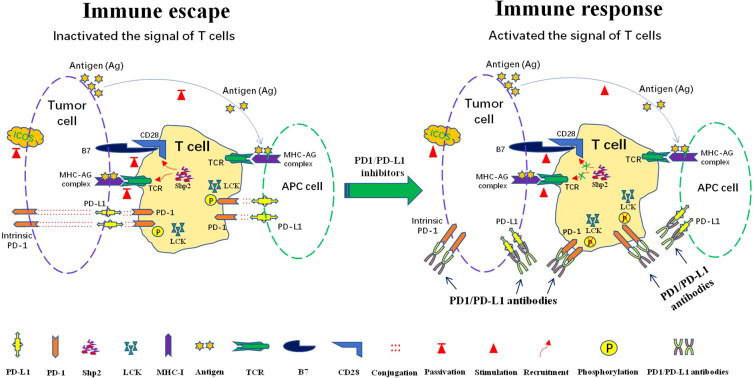
Mechanisms of the response to anti-PD1/PD-L1 immunotherapy: PD-L1 is expressed in tumor cells and antigen presenting cells (APCs). PD-1 is mainly expressed in T cells, some tumor cells also express intrinsic PD-1. Immune escape occur after interaction of PD-1 and PD-L1. PD-1 can be phosphorylated by LCK to recruit tyrosine phosphatase Shp2, consequently inactivating CD28 and T cell receptor (TCR) function and signaling pathway, thus attenuating the activation signal of T cells and causing immune escape. Lck kinase activity is required to maintain PD-1/Shp2-mediated inhibitory signaling. The intervene of PD-1/PD-L1 immunocheckpoint inhibitors can effectively block the interaction between PD-L1 and PD-1, which in turn blocks the recruitment of SHP-2 and reactivates T cells signal for immune function.

At present, the immunotherapy mechanisms of anti-PD-1/PD-L1 antibody have been relatively clear. The activation of T cells relies mainly on dual signals. The first signal consists of the binding of MHC-presenting antigen to T cell receptor (TCR). The second signal constitute by co-stimulatory and co-inhibitory signals (15). The interaction between PD-1 on T cells and PD-L1 on tumor cells or APCs can effectively inhibit T cell activation and even cause T cell apoptosis, decreased cytokine production, t-cell lysis and induction of tolerance to antigen, thus making the tumor escape from the immune surveillance (16). PD-1/PD-L1 inhibitors respectively bind to PD-1 or PD-L1 to prevent the interaction between PD-1 and PD-L1, then the recognition and killing effect of immune cells is restored and the immune escape of tumor cells is avoided (**Figure 1**).

PD-1 activation significantly inhibits TCR signaling, CD28 co-stimulatory signaling and inducible T cell co-stimulator(ICOS) signaling (17–19). Recent studies have suggested that after activation by its ligand PD-L1, PD-1 is phosphorylated by protein tyrosine kinase Lck to recruit tyrosine phosphatase Shp2 (Src homologous phosphatase 2), followed by dephosphorylation of TCR and CD28, and subsequent inhibition of T cell-associated signaling (20–23)(**Figure 1**). When PD-1/PD-L1 immune checkpoint inhibitors intervene, the intramembrane motif of PD-1 cannot be phosphorylated by lymphocyte-specific protein tyrosine kinase (LCK), resulting in the failure of cell recruitment to SHP-2. TCR and CD28 dephosphorylation is blocked, which leads to efficient delivery of activation signals to downstream proteins and signaling pathways, ultimately stimulating T cell proliferation and differentiation. Eventually, the immune function of T cells is effectively performed (24) (**Figure 1**). Interestingly, some tumor cells also express intrinsic PD-1 to promote the occurrence and development of tumors independent of adaptive immunity. PD-1 checkpoint inhibitors can also block the binding of intrinsic PD-1 and PD-L1 to inhibit tumor growth (25, 26) (**Figure 1**).

Tumor cells evolve themselves to lose the ability to present tumor antigens in order to avoid recognition by cytotoxic T cells and APCs (27). Recent studies showed that major histocompatibility complex class-I and -II (MHC-I and MHC-II) were required for tumor antigen presentation and immunosurveillance (28–30). In many malignancies, downregulation of MHC-I/II is associated with immunosuppression, metastatic progression and poor prognosis, as well as predict anti-PD-1/PD-L1 therapy response (31). Researchers have attempted to find ways to upregulate MCH-II expression in tumor cells with a view to improve the response rate to PD-1/PD-L1 immunotherapy. They found that epigenetic and ERK signaling cascades were effective in suppressing the expression of intrinsic MHC II in non-small cell lung cancer (32). Therefore, the combined blocking strategy for these pathways may generate a novel positive response to PD-1/PD-L1 immune checkpoint therapy in human cancers. In addition, lung epithelial MHC-II was needed for surface expression of PD-L1 (33). The results of a clinical study showed that recurrent or metastatic nasopharyngeal carcinoma with high expression of both MHC-II and PD-L1 responded better to treatment with camrelizumab (anti-PD-1) (34). In conclusion, the above results suggest that MHC-II and PD-L1 influence each other not only in expression but also in function for the treatment of PD-1/PD-L1 Immune-checkpoint inhibitors in human cancer.

## Mechanisms of PD-1/PD-L1 inhibitors resistance

**Figure 2:**
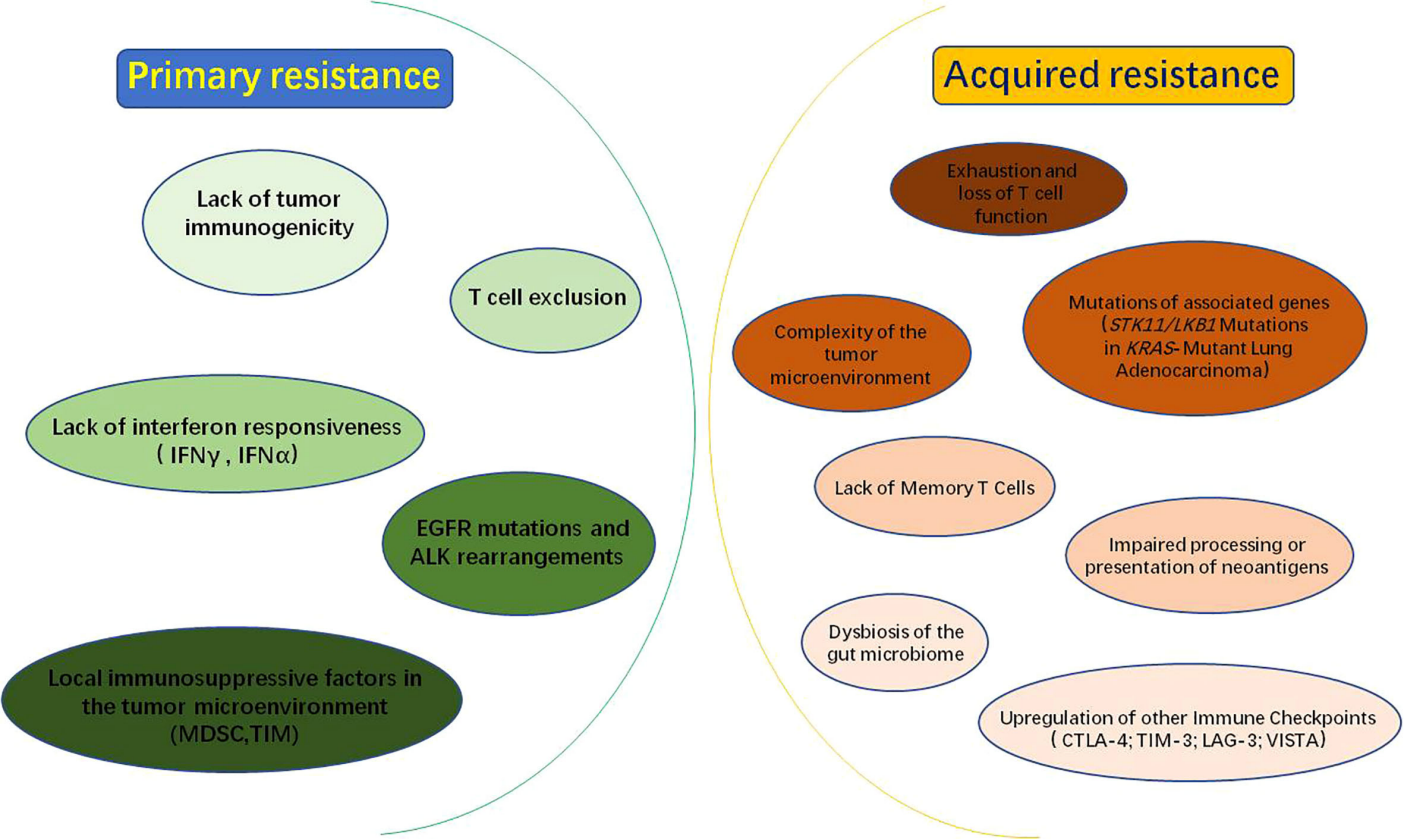
Mechanisms of PD-1/PD-L1 inhibitors resistance: PD-1/PD-L1 inhibitor resistance is divided into primary resistance and acquired resistance. Mechanisms of primary resistance include lack of tumor immunogenicity; T-cell rejection; lack of interferon responsiveness, such as IFNγ (interferon Gamma) and IFNα (interferon alpha); EGFR (epidermal growth factor receptor) mutations and ALK (anaplastic lymphoma kinase) rearrangements; local immunosuppressive factors within the tumor microenvironment, such as MDSC (myeloid-derived suppressor cell) and TIM (tumor-infiltrating myeloid cell). While, the mechanisms of acquired resistance may be related to the following factors: exhaustion and loss of T cell function; impaired processing or presentation of neoantigens; complexity of the tumor microenvironment; mutations in associated genes, such as STK11/LKB1; dysbiosis of the gut microbiome; lack of Memory T Cells and upregulation of other Immune Checkpoints, such as CTLA-4(cytotoxic T-lymphocyte antigen-4), TIM-3(T cell immunoglobulin and mucin domain-containing molecule-3), LAG-3(lymphocyte activation gene-3) and VISTA(V-domain Ig suppressor of T cell activation).

In recent years, immune checkpoints blockade therapy targeting the PD-1/PD-L1 axis has pushed tumor immunotherapy to a new revolutionary-like milestone and achieved surprising therapeutic effects in a variety of malignancies. However, most patients have developed resistance to PD-1/PD-L1 inhibitors, which severely limits its application and becomes a serious clinical problem that cannot be ignored in this field. Therefore, it is urgent to deeply reveal the molecular mechanism of immune checkpoint inhibitor resistance and improve the response rate of patients to PD-1/PD-L1 immunotherapy. Herein, we have summarized the molecular mechanisms of resistance to common PD-1/PD-L1 immune checkpoint inhibitors (**Figure 2**).

The resistance to PD-1/PD-L1 inhibitors includes primary resistance and acquired resistance. Primary resistance is defined as patients who have never shown clinical response or stable disease when using PD-1/PD-L1 blockade. The mechanism of primary resistance includes lack of tumor immunogenicity (35), T cell exclusion (36), lack of interferon responsiveness (37), epidermal growth factor receptor (EGFR) mutations and anaplastic lymphoma kinase(ALK) rearrangements (38), local immunosuppressive factors in tumor microenvironment (39) and other factors (**Figure 2**). While acquired drug resistance means that PD-1/PD-L1 inhibitors show a durable and effective response at the beginning of treatment, but therapeutic effect of inhibitors is significantly diminished or non-responsive after a period of treatment for some patients (40). The mechanisms may be closely associated with exhaustion and loss of T cell function (41–43), impaired processing or presentation of neoantigens (44), complexity of the tumor microenvironment (45), mutations in associated genes (46), dysbiosis of the gut microbiome (47), lack of Memory T Cells (48) and upregulation of others immune checkpoints (49) (**Figure 2**).

Tumor cells may interact closely with stromal cells, immune cells, other suppressive immune checkpoints and cytokines in the surrounding environment, thus protecting them from detection and elimination by immune surveillance (45). In general, T lymphocytes accomplish immune clearance of tumors by recognizing specific antigens on the surface of tumor cell membranes, thereby killing tumor cells. Therefore, effective tumor-specific antigen is an important factor for the efficacy of immune response. If the structure of the specific antigen is similar to the immune tolerance antigen or autoantigen, resulting in the inability of APCs to recognize and initiate T-cell activation, thus acquired resistance may developed (50, 51). In addition, some tumors cause a decrease of normal mature dendritic cells (DCs) and an increase in the number of immature DCs by secreting certain suppressors, such as IL-10 and VEGF. When tumors recruit these immature DCs, effector T cells are not effectively activated during antigen presentation (24, 52). These patients will fail to generate an effective immune response with PD-1/PD-L1 blockers, resulting in drug resistance and immune escape.

## Main clinical trials and outcomes for PD-1/PD-L1 inhibitors in the treatment of human cancers

In recent years, a large number of clinical trials have been conducted with PD-1/PDL1 Immune-checkpoint inhibitors, and their therapeutic effects have been evaluated from different perspectives, including overall survival (OS), objective response rate (ORR) and medium progression-free survival (PFS), respectively (53–55). Herein, we reviewed some clinical trials of PD-1/PDL1 immunocheckpoint inhibitors (**Table 1**). We found that the same inhibitor has completely different therapeutic effects and responsiveness against different cancers **(** **Table 1****).** This will provide better reference for the selection of PD1/PD-L1 inhibitors for different cancers in future clinical practice.

**Table 1:** Clinical trials of PD-1/PD-L1 inhibitors in human cancers.

Inhibitors	Style	Cancers	Trial number	N	OS	ORR		PFS	References
Pembrolizumab	PD-1	Gastric cancer	NCT02589496	61	N/A	85.7% in microsatellite instability-high mGC	100% in Epstein-Barr virus-positive mGC	N/A	(56)
		Pancreatic cancer	NCT02054806	475	N/A	0.0% to 14.2%		1.7 months 1.5 to 2.9 months)	(57)
		Small-cell lung cancer			N/A	33% (15.6% to 55.3%)		N/A	
		Thyroid cancer			N/A	N/A		6.8 months (1.9 to 14.1 months)	
		Non-Small-Cell Lung Cancer	NCT02142738	305	N/A	N/A		10.3 months	(58)
		Breast cancer	NCT02447003	84	18 months			2.1 months	(59)
		Non-small-cell lung cancer	NCT02775435	559	15.9 VS 11.3 months (pembrolizumab-combination group VS placebo-combination group)	N/A		( 6.4 VS 4.8 months pembrolizumab- combination group VS placebo-combination group)	(60)
		Gastric cancer	NCT01848834	39	N/A	22%		N/A	(61)
		Non-small-cell lung cancer	NCT01295827	495	12 months	19.40%		3.7 months	(62)
		Melanoma	NCT01295827	655	N/A	8%, 12%, 22%, 43%, 57%, and 53% for MEL scale of 0, 1. 2, 3, 4 and 5		N/A	(1)
		Hepatocellular carcinoma	NCT02702414	104	N/A	17%		N/A	(63)
		Malignant pleural mesothelioma	NCT02054806	25	N/A	20%		N/A	(64)
Nivolumab	PD-1	Advanced hepatocellular carcinoma	NCT01658878	262	N/A	20%		N/A	(63)
		Hodgkin Lymphoma	NCT02181738	80	N/A	66.30%		N/A	(65)
		ovarian cancer	NCT02873962	38	N/A	40% in platinum-sensitive and 16.7% in platinum- resistant participants		8.1 months	(66)
		Follicular lymphoma	NCT01592370	10		40%		N/A	(67)
		Diffuse large B- cell lymphoma		11	N/A	36%		N/A
		Peripheral T-cell lymphoma		5	N/A	40%		N/A
		Melanoma	NCT01844505	945	N/A	N/A		11.5 VS 2.9 months(nivolumab plus ipilimumab group VS ipilimumab alone group)	(68)
Atezolizumab PD-L1	PD-1	Triple-Negative Breast Cancer	NCT02425891	451	21.3 VS 17.6 months(atezolizumab plus nab- paclitaxel group VS placebo plus nab- paclitaxel group)	N/A		7.2 VS 5.5 months(atezolizumab plus nab-paclitaxel group VS placebo plus nab- paclitaxel group)	(69)
		Non-small-cell lung cancer	NCT02008227	1225	15.7VS 10.3 months (atezolizumab group VS docetaxel group)	N/A		N/A	(70)
Toripalimab	PD-1	Alveolar soft part sarcoma	NCT02836834	12	34.7 months	22.70%		5.7 months	(71)
		Lymphoma		11	N/A	90.90%		8.3 months
		Non-Small Cell Lung Cancer	NCT03301688	41	13. 8 months among 28 patients included in the response and survival analysis	N/A		2.8 months among 28 patients included in the response and survival analysis	(72)
Durvalumab PD-L1		Head and neck squamous cell carcinoma	NCT02207530	112	7.1 months	N/A		2. 1 months	(12)
		Non-Small Cell Lung Cancer		406	13.- months; 3.4 VS16.2 months (HPD VS Non-HPD)	18.90%		2.1 months	(73)
Avelumab	PD-L1	Metastatic breast cancer	NCT01772004	168	N/A	3.0% overall	5.2% in patients with TNBC	N/A	(74)
Tislelizumab	PD-1	Hodgkin lymphoma	NCT03209973	70	N/A	87.10%		74.5% (9-month progression-free survival rate)	(75)
Camrelizumab	PD-1	Hodgkin lymphoma	NCT03155425	75	N/A	76.00%		N/A	(76)
GLS-010	PD-1	Hodgkin lymphoma	NCT03713905	24	N/A	87.50%		N/A	(77)
		Peripheral NK T lymphoma		14	N/A	21.4%		N/A

N/A, Not Applicable.

## Immune-related adverse events caused by PD-1/PD-L1 inhibitors in the treatment of human cancers

Over the past few decades, cancer immunotherapies represented by PD-1/PD-L1 immune checkpoint inhibitors have changed the landscape of cancer treatment. However, this has also inevitably led to some immune-related adverse events (irAEs), and these irAEs are usually characterized by long duration and delayed onset (58, 78–86). In this study, we have reviewed some of the common immune-related adverse events associated with PD-1/PD-L1 immune checkpoint inhibitors for cancer treatment (**Table 2**).

**Table 2:** Immune-related adverse events caused by PD-1/PD-L1 inhibitors in human cancers.

Inhibitors	Styles	Cancers	Total number of patients (N)	Immune-related adverse events (Number of events/total number of patients, n/N)					References
				Rash	Hypothyroidism	Elevated AST	Colitis	Pneumonitis	
Nivolumab	PD-1	Squamous-CellCarcinoma	236	18/236	9/236	2/236	N/A	5/236	(80)
		Hepatocellularcarcinoma	48	11/48	N/A	10/48	N/A	N/A	(63)
		Ovarian Cancer	38	4/38	N/A	10/38	N/A	4/38	(66)
		Diffuse Large B Cell lymphoma	121	6/121	N/A	N/A	N/A	N/A	(87)
		Hodgkin lymphoma	80	1/80	N/A	2/80	N/A	1/80	(65)
Pembrolizumab	PD-1	NSCLC	1034	29/1034	28/1034	10/1034	N/ A	16/1034	(81)
		NSCLC	154	6/154	14/154	N/A	3/154	9/154	(58)
		Gastric cancer	39	N/A	4/39	N/A	N/A	1/39	(61)
		Hepatocellularcarcinoma	104	10/104	6/104	7/104	N/A	1/104	(88)
		Advanced urothelial cancer	266	23/266	19/266	N/A	N/A	N/A	(82)
Atezolizumab	PD-L1	NSCLC	144	N/A	N/A	6/144	2/144	4/144	(83)
		Hepatocellularcarcinoma	58	6/58	N/A	6/58	N/A	N/A	(84)
		NSCLC	609	N/A	N/A	N/A	2/609	6/609	(70)
		Urothelial carcinoma	119	6/119	8/119	4/119	N/A	N/A	(85)
Tislelizumab	PD-1	Solid tumors	451	61/451	N/A	23/451	6/451	13/451	(86)
Toripalimab	PD-1	NSCLC	41	6/41	3/41	5/41	N/A	1/41	(72)
		Gastric Cancer	58	5/58	7/58	7/58	N/A	N/A	(89)

N/A, Not Applicable.

## PD-1/PD-L1 and inhibitors in human solid cancers

### Lung cancer

Lung cancer is the most common cancer and the leading cause of cancer death worldwide. Fortunately, the advent of immune checkpoint inhibitors has improved the outlook for patients with advanced lung cancers. Tumor immunotherapy targeting PD-1/PD-L1 have revolutionized the treatment of lung cancer (70, 72, 90).

Elevated PD-L1 expression correlates with higher efficacy of immunotherapy, implying that PD-L1 has high predictive value as a cancer biomarker (62, 91). The anticancer efficacy of PD-L1 inhibitors is significantly better than that of chemotherapy in advanced non-small cell lung cancer (NSCLC) patients with high PD-L1 expression (58),as well as in patients with previously untreated metastatic squamous NSCLC (60). Remarkably, PD-L1 expression may also be induced by chemotherapy or targeted therapies (92). Therefore, if PD-L1 protein expression is to be used as a biomarker to guide immunotherapy, fresh specimens may need to be collected after other treatments and before the start of immunotherapy to assess PD-L1 expression. Moreover, tumor microenvironment plays an important role in the anticancer effect of PD-1/PD-L1 immunocheckpoint inhibitors. Recent studies have shown that low-dose apatinib (VEGFR2-TKI) significantly improves the therapeutic effect of PD-1/PD-L1 inhibitors by modulating the tumor microenvironment, delaying tumor growth, reducing the number of metastases and prolonging survival in mouse models (93).

In patients with lung cancer, PD-1 and PD-L1 can be detected not only in tissues but also in the serum and plasma of patients (94–96). The clinical diagnosis and prevention of soluble PD-1 and PD-L1 lung cancer in blood is of great importance because blood samples are easily available and easily detectable (96). By detecting the expression levels of PD-1 and PD-L1 in the blood of lung cancer patients, the drug treatment regimen of PD-1 and PD-L1 can be formulated based on the test results, and the patients’ response to immunotherapy can be further assessed. The purpose of individualized treatment of lung cancer is absolutely achieved.

Unfortunately, although many patients have achieved long-term survival benefits with PD-1/PD-L1 inhibitors, some patients have experienced rapid tumor progression after immunotherapy, known as hyperprogressive disease (HPD) (97, 98). In pretreated NSCLC patients, HPD is more common with PD-1/PD-L1 inhibitors compared to chemotherapy, and patients treated with PD-1/PD-L1 inhibitors are also associated with a high metastatic burden and poor prognosis (73). Currently, the combination of PD-1/PD-L1 immune checkpoint inhibitors with other antitumor agents has become an important treatment strategy. For example, the combination of pembrolizumab antibody (anti-PD-1) with carboplatin plus paclitaxel reduced the risk of death in advanced NSCLC. Atezolizumab antibody(anti-PD-L1) combined with carboplatin plus paclitaxel also improved the treatment outcome in advanced NSCLC (99).

The rapid development of anti-PD-1/PD-L1 inhibitors for advanced NSCLC has greatly improved patient prognosis. However, the vast majority of NSCLC patients are ineffective to PD-(L)1 blockade. Therefore, more clinical trials are required to explore immunomodulatory pathways in an effort to enhance non-responders or hyposensitive individuals to achieve desired therapeutic outcomes. In addition, understanding the mechanisms of resistance to immune checkpoint inhibitors will help determine combination therapy strategies for advanced lung cancer.

### Breast cancer

In the past few years, anti-PD-1/PD-L1 antibodies have shown promising therapeutic effects, and great anti-tumor effects have been observed when used alone or in combination with conventional treatment. However, immunotherapy is still rarely used in the treatment of breast cancer. In fact, breast cancer is generally thought to have a weaker immunogenicity than other types of tumors (100).

Encouragingly, specific PD-1/PD-L1 antibodies can effectively block PD-1 or PD-L1 in breast cancer. Especially, metastatic triple negative breast cancer (mTNBC) shows a potential response to PD-1/PD-L1inhibitors. For example, pembrolizumab antibody(anti-PD-1) has significant antitumor activity and safety in patients with PD-L1-positive mTNBC (16, 59). Moreover, atezolizumab antibody (anti-PD-L1) effectively prolong progression-free survival in patients with mTNBC, and paclitaxel enhanced the therapeutic effect of atezolizumab (69). In addition, patients with metastatic breast cancer (MBC) were treated with avelumab (anti-PD-L1) for 2-50 weeks and followed up for 6-15 months. The results showed that the objective response rate (ORR) was significantly increased in patients with PD-L1 positive tumor-associated immune cells, which suggested that PD-L1 is associated with higher response rates to avelumab in patients with MBC (74).

However, it has also been suggested that PD-1 inhibitors are less effective in mTNBC and that better strategies should be adopted to make the tumor microenvironment more sensitive to PD-L1 inhibitors. For example, short-term adriamycin and cisplatin may induce a more favorable tumor microenvironment in mTNBC and increase the anticancer effect of PD-1 blockers (101). In the past, chemotherapy was the standard first-line treatment for mTNBC, but the efficacy was not satisfactory. Recent study have found that the combination of PD-1/PD-L1 Immune-checkpoint inhibitors and chemotherapy may be a new promising clinical paradigm for the treatment of triple-negative breast cancer (102).

Moreover, PD-L1 expression is associated with high-risk clinicopathological parameters and poor prognosis in patients with primary breast cancer (PBC). A meta-analysis, including 47 studies with a total of 14,367 PBC patients, suggested that PD-L1 high expression associates with large tumor size, histologic grade, Ki-67 high level, ER and PR negative, TNBC subtype and shorter survival time (103). In addition, the expression of PD-L1 in breast cancer stem cells has attracted interest in recent years, and studies have found a significant increase in PD-L1 protein in breast cancer stem cells; therefore, targeting PD-L1 in stem cells may become a new promising therapeutic strategy for breast cancer (104). In any case, PD-1 and PD-L1 have been increasingly studied in breast cancer in recent years, and PD-1/PD-L1 immunocheckpoint inhibitors have shown promising applications in the treatment of breast cancer.

### Gastric cancer

Gastric cancer (GC) is a common malignancy and the third leading cause of cancer death worldwide. The 5-year survival rate of patients with advanced gastric cancer was only 5% and 20%, and the median overall survival rate was 10 months. Advanced gastric cancer has a poor prognosis and limited therapeutic options (89, 105, 106). Therefore, it is urgent to explore some new molecular targets and treatments.

Clinical studies have confirmed the efficacy of programmed cell death 1 (PD-1)-targeted therapy for patients with metastatic GC. For example, the anti-PD-1 antibody pembrolizumab has promising anti-tumor activity and manageable toxicity in the treatment of patients with recurrent or metastatic GC (61). Moreover, anticancer effect and responsiveness of pembrolizumab are closely related to PD-L1 expression. Pembrolizumab has a significantly higher ORR in PD-L1-positive GC than in PD-L1-negative GC (56).

It is well known that autophagy is a highly conserved homeostasis process that plays a key role in tumor formation, cell survival, cell metabolism, immune response and tumorigenesis (107–109). Recent studies have shown that autophagy is highly associated with the expression level of PD-L1. Autophagy inhibition increases the expression of PD-L1 in GC, autophagy-related protein LC3 expression is also positively correlated with PD-L1 in primary GC (110). Therefore, autophagy may be closely related to anti-PD-1/PD-L1 immunotherapy in human GC.

Although anti-PD-1/PD-L1 immunotherapy is widely recognized in the clinical practice of gastric cancer, some studies have questioned that single PD-1/PD-L1 inhibitor do not result in relative improvements in OS and PFS compared with chemotherapy in patients with advanced GC or gastroesophageal junction cancer. However, they also determined that PD-1/PD-L1 inhibitors appear to enhance antitumor activity in patients with advanced gastric junction cancers (111). Therefore, further randomized clinical trials are needed to confirm those findings.

### Angiosarcoma

Angiosarcoma (AS) is rare malignant endothelial-cell tumors of vascular or lymphatic origin, and is among the most aggressive subtypes of soft-tissue sarcomas (112, 113). In recent years, immunotherapy targeting PD-1/PD-L1 has become a hotspot in the treatment of AS (114, 115). A recent study analyzed PD-L1 expression levels in angiosarcomas at different sites in humans and showed that PD-L1 was abnormally expressed in about 66% of the samples (116). In addition, a 63-year-old male patient with nasal AS that received pembrolizumab 2 mg/kg every 21 days for 13 cycles had no new tumor progression during the 8 months after therapy, which suggested that PD-1/PD-L1 immune checkpoint inhibitors have significant efficacy against angiosarcomas (117).

Cutaneous angiosarcoma (CAS) is the most common form of AS. Positive PD-L1 expression predicts worse outcome in CAS (118). Malignant progression and prognosis of CAS are closely associated not only with high expression of PD-L1, but also with the presence of tumor-infiltrating lymphocytes (TILs) (114). Since high PD-L1 expression is closely related to the progression of CAS, it is also critical to explore upstream regulators that can increase PD-L1 expression. Recent study revealed that PD-L1 expression was closely associated with atypical protein kinase C lambda/iota (aPKCλ). Inhibition of aPKCλ expression in HUVECs significantly reduced the expression of PD-L1 (119). Therefore, the combination of immune checkpoint inhibitors and aPKC inhibitors may be a potential therapeutic strategy for patients with CAS.

With the development of genomic sequencing technology, treatment strategies for advanced diseases have advanced significantly. Whole genome sequencing (WGS) can provide valuable information for treatment of PD-1/PD-L1 Immune-checkpoint inhibitors in the treatment of AS. For example, patients with metastatic AS who underwent WGS analysis were found to have hypermutated tumor characteristics associated with a positive response to PD-1 Immune-checkpoint inhibitors (120). Subsequently, corresponding scheme was established for the patient to receive the anti-PD-1 antibody pembrolizumab, and the metastases almost completely disappeared after 4 weeks therapy (120). Taken together, PD-1/PD-L1 expression is related to AS progression, and growing evidence suggests that the treatment with PD-1/PD-L1 immunocheckpoint inhibitors may be a promising strategy for AS patients.

### Prostate cancer

Prostate cancer (PC) remains the most commonly diagnosed malignant disease in men worldwide (121). At present, PD-1/PD-L1 immunocheckpoint inhibitors have brought significant clinical benefits to some patients with PC. Further study will help guide the development of immunotherapy for advanced PC (122, 123). Patients with high density of PD-1+ lymphocytes were at significantly higher risk of clinical failure, and it was positive association between a high density of PD-1+ lymphocytes and worse clinical failure-free survival (124). Pembrolizumab(anti-PD-1) achieve durable objective responses in a group of severely pretreated patients with advanced PD-L1 positive PC (125). Moreover, recent study suggested that the combination of PD-1/PD-L1 checkpoint inhibitors and radiotherapy was a promising strategy for treating PC (126). Interestingly, PD-1/PD-L1 inhibitors significantly enhanced the efficacy of SA-GM-CSF surface-modified tumor vaccines against PC, which may be a new application for PD-1/PD-L1 inhibitors in the treatment of PC (127). In addition, PD-1/PD-L1 inhibitors had potential of lasting response to microsatellite instability-high (MSI-H) or defective mismatch repair (dMMR) molecular phenotype of prostate cancer (128). Nevertheless, more relevant studies may be needed to confirm this issue.

However, it was reported that PD-L1/PD-1 blockade had a poor effect in PC, due to the low immunogenicity of PC. Early clinical trials confirmed that patients with metastatic castration-resistant prostate cancer (mCRPC) did not significantly respond to PD-1 inhibitors, and they believed that loss of PTEN is responsible for upregulation of PD-L1, followed by constituting innate immune resistance (129). However, subsequent studies revealed that high PD-L1 expression is not significantly related to the loss of PTEN, but rather to the regulation of inflammatory cytokines (130).

All in all, although PD-1/PD-L1 Immune-checkpoint inhibitors have made revolutionary breakthroughs in the treatment of a wide range of human cancers, only a small percentage of prostate cancer patients have achieved significant clinical benefit. However, many experts support that we should still encourage clinical trials with PD-1/PD-L1 inhibitors in PC patients and exploring the mechanisms of PD-1/PD-L1 inhibitors resistance will optimize treatment options and guide the next steps in immunotherapy for PC.

### Colorectal carcinoma

Colorectal cancer (CRC) is one of the most common neoplasms accompanied by a high rate of morbidity and mortality, immune checkpoint molecules have been identified as a novel treatment for CRC, such as PD-1 and PD-L1 (131, 132).

Currently, several clinical studies gave the clinical conclusions for PD-1 inhibitors in CRC with dMMR and MSI-H (133, 134). In a non-randomized phase II clinical trial enrolling 74 metastatic CRC(mCRC) patients with dMMR/MSI-H, patients were treated with nivolumab antibody (anti-PD-1) with relatively satisfactory clinical results. The results showed that ORR is 31.1% was achieved, disease control longer than 12 weeks was achieved in 69% of patients, twelve months PFS was 50.4% and 12 months OS was 73.4% (135). Immune checkpoint inhibitors have achieved clearer therapeutic effect in mCRC with dMMR or high microsatellite instability (MSI-H) (133, 136). However, patients with proficient mismatch repair(pMMR) or microsatellite stable (MSS) tumors have not gained enough benefit from immunotherapy (137). This may be related to the higher expression of PD-1 and PD-L1 in dMMR tumors compared to pMMR tumors (138). For pMMR CRC patients with poor responsiveness to immunotherapy, recent study has demonstrated a significant synergistic inhibitory effect of pembrolizumab(anti-PD-1) combined with ibrutinib (139). The combination of pembrolizumab and azacitidine for chemotherapy-refractory mCRC has also achieved a safe, tolerable and positive clinical efficacy (140). Moreover, the chemotherapy agent FOLFOXIRI plus bevacizumab is known to increase the immunogenicity of pMMR or MSS tumors. Importantly, the addition of atezolizumab(anti-PD-L1) to the first-line FOLFOXIRI plus bevacizumab significantly improve progression-free survival in patients with previously untreated metastatic colorectal cancer (141). Taken together, the application of PD-1/PD-L1 inhibitors in combination with other antitumor agents will bring light to the treatment of refractory or metastatic CRC.

It is generally believed that PD-1/PD-L1 cause immune escape from tumors by inhibiting tumor immune processes. Reduction of T cell cytotoxicity may be one of the key mechanisms of tumor PD-L1-induced inhibition of antitumor immunity. A recent study suggested that PD-L1 expressed in CRC significantly inhibited the cytotoxicity of CD8^+^ T cells, which led to tumor immune escape (142). In addition, tumor immune escape may be caused by upregulation of tumor cell infiltrating immune cells (TIICs) or their ligands at suppressive immune checkpoint in CRC, such as PD-1, PD-L1 and CTLA-4. Changes in DNA methylation patterns and enrichment of methylated histone markers in promoter regions may be the main reasons for upregulation of immune checkpoint (ICs) in CRC (143). Furthermore, a study aimed at analyzing the prognostic value of PD-L1 in CRC cells and tumor cell infiltrates (TILs) revealed that high expression of PD-1 and PD-L1 was associated with a better prognosis in colorectal cancer patients and TILs-PD-1 may be an independent prognostic factor for OS and disease-free survival (DFS) in CRC patients (144).

Anyway, PD-L1 inhibitor-based immunotherapy is considered a promising approach for targeting colorectal cancer. The binding of PD-L1 and PD-1 in tumor cells or tumor microenvironment induces immunosuppressive signals that reduce T cell proliferation and lead to tumor immune escape.

### Hepatocelullar carcinoma

Hepatocelullar carcinoma (HCC) is the third leading cause of cancer death and the sixth most common malignancy worldwide (145). Recent study showed that PD-1/PD-L1 expression played an important role and interacted with CD8^+^ T-cell immune responses to regulate the immune homeostasis and prognosis of HCC patients (146). Recent studies revealed that amplification or high expression of PD-L1 was significantly and independently associated with poor survival in HCC patients, which confirmed that the PD-1/PD-L1 axis is a promising potential target for HCC immunotherapy (147).

Recent studies have shown that immunocheckpoint inhibitor therapy significantly improves the overall survival of HCC patients (148, 149). For example, some PD-1 inhibitors, such as pembrolizumab and nivolumab, were effective and well tolerated in patients with advanced HCC (63, 88). PD-L1 mediated the growth inhibition of herbal medicines baicalin and flavonol on HCC by decreasing STAT3 activity, thereby restoring the anti-cancer sensitivity of T cells (150). More importantly, anti-PD-1/PD-L1 antibodies combined with other therapies was considered an effective strategy for the treatment of HCC (151). In addition to binding to cell membranes, PD-1/PD-L1 could also dissociate in the blood of patients and is called as soluble PD-1/PD-L1 (94, 152). Recent studies have shown that high expression of soluble PD-L1 was significantly associated with an increased risk of death (153, 154). Furthermore, soluble PD-1 and PD-L1 were independent prognostic factors with opposite roles in predicting disease-free survival (DFS) and overall survival (OS) in HCC patients (155).

Currently, clinical trials about the application of PD-1/PD-L1 checkpoint inhibitors in HCC are underway or completed, some of which have shown promising therapeutic expectations. Nonetheless, the clinical benefits for other patients are not satisfactory. Comprehensive predictive biomarkers are necessary to identify HCC patients who are more likely to respond to immunosuppression and thus guide clinical therapy strategies.

### Bladder cancer

Bladder cancer (BC) is the most common cancer of the human urinary system, with poor prognosis and high recurrence rate (156, 157). Immunotherapy based on PD-1/PD-L1 immunecheckpoints has been approved and successfully performed in the treatment of BC (158–160). Recent studies have identified significant differences in the expression levels of PD-1 and PD-L1 between higher-grade BC and lower-grade BC. The expressions of PD-1 and PD-L1 in higher-grade BC are higher than those in lower-grade BC. Therefore, PD-1 and PD-L1 may be important biomarkers related to the pathological grading of BC and play a mediating role in the progression of BC (161). Moreover, PD-L1 may be novel combined biomarkers for predicting tumor invasitivity and immune checkpoint response in BC (162). Taken together, PD-1/PD-L1 are closely associated with tumorigenesis, treatment and prognosis of human BC.

PD-L1 positive BC patients with heavily pretreated have shown a manageable safety profile and meaningful clinical outcomes after treatment of durvalumab antibody (Anti-PD-L1), ORR was 46.4% and responses were ongoing in 12 of 13 responding patients (163). Another study evaluated the safety and antitumor activity of avelumab (Anti-PD-L1) in patients with metastatic urothelial BC. Results suggested that patients achieved a median progression-free survival of 11.6 weeks, median OS of 13.7 months and 12-months OS rate of 54.3% (164). There were many other similar clinical studies that demonstrate that PD-1/PD-L1 Immune-checkpoint inhibitors are well associated with durable responses and prolonged survival in metastatic urothelial BC. Recent studies revealed that the expression and function of PD-L1 in BC were closely related to autophagy. PD-L1 could be upregulated by autophagy-related proteins (e.g ATG7), ultimately enhancing the stem cell-like properties and invasive capacity of BC cells (165). Therefore, PD-1/PD-L1 Immune-checkpoint inhibitors combined with autophagy inhibitors may be a promising therapeutic approach for human BC. In addition, anti-PD-1/PD-L1 immunetherapy combined with radiotherapy has significant local and distal synergistic anticancer effects (166).

Overall, PD-1/PD-L1 Immune-checkpoint inhibitors have shown promising results in terms of clinical efficacy in patients with advanced and metastatic BC. However, more additional data is urgently needed for the evaluation of reliability and safety of anti-PD-1/PD-L1 treatment. These exciting advances will bring more benefit and hope for BC patients.

### Ovarian cancer

Ovarian cancer(OC) is the seventh most common cancer and the eighth leading cause of cancer death in women worldwide (167). Recent studies have shown that the application of PD-1/PD-L1 inhibitors in the treatment of OC has attracted extensive attention of researchers (168–170). PD-1 and its ligand were significantly expressed in tumor cells and immune system cells of OC patients (171).

Recent studies claimed that PD-1/PD-L1 immune checkpoint inhibitors do not perform well in the treatment of recurrent epithelial OC (172, 173). However, well efficacy was achieved by the combination of PD-1/PD-L1 inhibitor (Nivolumab) and anti-angiogenic drug (Bevacizumab). Their interaction may exert synergistic effects by modulating the microenvironment (66). In addition, combination therapy with PARP and immune checkpoint inhibition has yielded encouraging results in ovarian cancer (174, 175). Taken together, combination therapy may be an effective strategy for the treatment of OC, offering a potential therapeutic opportunity for OC.

Although combined immunotherapy have evolved rapidly in the treatment of OC and have been successfully applied, some emerging issues need to be addressed in clinical practice, such as the dose and sequence of optimal synergy, differences in immunotherapy response across OC subtypes and possible side effects of the interaction of two medicines. Therefore, we appeal that more clinical trials of combination immunotherapy should be performed to obtain relevant clinical data, including efficacy, stability and immune-related adverse effects. so that the combined immunotherapy will be more widely applied in the treatment of OC in the near future.

### Pancreatic cancer

Pancreatic cancer (PC) is one of the leading causes of cancer death worldwide. In the last two decades, the number of pancreatic cancer patients diagnosed each year has doubled worldwide (176). PD-L1 was identified as a novel maker of prognosis in patients with PC, and the up-regulation of PD-L1 was found in human PC tissues (177). PD-L1 is involved in the regulation of PC stemness, epigenetic mechanisms and metastasis. Blocking PD-1 significantly inhibited the PC growth by enhancing INF-γ production and decreasing IL-10 production in a mouse model (178). Therefore, PD-1/PD-L1 expression and related signaling may play an important role in the progression of PC.

In recent years, although PD-1/PD-L1 immunecheckpoint inhibitors have been rapidly developed in the treatment of various cancers. However, the outcomes of PD-1/PD-L1 immunecheckpoint inhibitors monotherapy are not satisfactory in PC (179). Currently, two main reasons are believed to be responsible for such failures. First, pancreatic cancer is inherently non-immunogenic. Second, immunosuppression due to high tumor burden is another reason for which PC cannot be treated by PD-1/PD-L1 blockade alone, immune escape of pancreatic tumors is closely related to the excessive development of immunosuppressive T cells (177). Therefore, the combination therapeutic strategies may bring new hope for the treatment of PC with PD-1/PD-L1 inhibitors. For example, the combination of anti-PD-1 inhibitory antibodies and anti-ox40 agonist antibodies decreased the proportion of T regulatory, and increased the number of memory CD4+ and CD8+ T cells, thereby attenuating the immune escape response and enhancing the anticancer effects of anti-PD-1 in PC (180). In addition, a study suggested that Anti-TNFR2 and anti-PD-L1 combination therapy significantly inhibited the growth of PC through relieving tumor immunosuppression and generating robust memory recall (181). Moreover, Anti-PD-1 antibody immunotherapy combined with gemcitabine significantly inhibited PC and liver metastasis by enhancing the immune response mediated by Th1 lymphocytes and M1 macrophages (182).

All in all, although anti-PD-1/PD-L1 inhibitors have rapidly developed as a priority of immunotherapy strategy for various cancers. However, the poor therapeutic outcomes were observed in the treatment of PC because of the particularity of pancreatic cancer, such as a high tumor burden, non-immunogenicity and immunosuppressive tumor microenvironment. The combination of anti-PD-1/PD-L1 immunotherapy with other anti-tumor agents that overcome these specific properties will significantly improve the therapeutic effect of anti-PD-1/PD-L1 immunotherapy in PC.

## PD-1/PD-L1 and inhibitors in human hematological malignancies

### Leukemia

Leukemia is the common name for several malignant diseases with an increasing number of white blood cells in the blood and/or bone marrow. Leukemia includes acute myeloid leukemia, chronic myeloid leukemia, acute lymphoblastic leukemia, chronic lymphocytic leukemia, and so on (183). Acute myeloid leukemia (AML) is the most common form of leukemia and hematological malignancy with a poor clinical prognosis and characterized by uncontrolled proliferation of hematopoietic stem cells in the bone marrow (184, 185).

Recent results has shown that high expression of PD-1 and PD-L1 was associated with poorer overall survival (OS) and clinical outcome in AML patients (186, 187). In addition, a clinical trial has shown encouraging response and overall survival rates for patients with relapsed/refractory (R/R) acute myeloid leukemia (AML) treated with nivolumab(anti-PD-1) and azacitidine, which suggested that nivolumab in combination with azacitidine appears to be a safe and effective treatment for AML (188).

However, the clinical response to PD-1/PD-L1 blockade varied in different AML patients (189). A recent study revealed that the majority of immune-checkpoint receptor genes were downregulated in bone marrow (BM)-infiltrating CD8+ T cells and partially in CD4+ T cells due to pathological chromatin remodeling via histone deacetylation. Therefore, the dysfunction of CD8+ T cells in AML was mainly due to pathological epigenetic silencing of activated IC receptors rather than due to signaling by immune inhibitory IC receptors (190). This may explain the limited role of PD-1/PD-L1 antibodies in AML patients. In conclusion, anti-PD-1/PD-L1 therapy may be a new immunotherapeutic strategy for AML. However, further studies are still necessary.

### Multiple myeloma

Multiple myeloma (MM) is a genetically heterogeneous clonal plasma cell disorder, which is the second most common malignancy in the hematological system (191, 192). The immune dysfunction is critical for the genesis of MM. The interaction of PD-L1 and PD-1 inhibited the body’s immune function and promoted immune escape by preventing tumor-reactive T cells from being activated and functioning (193). PD-L1 and PD-1 were higher on their tumor cells and T-cells in MM patients, respectively. MM cells with high PD-L1 expression effectively protected themselves against MM-specific t-cell killing, which could be reversed by anti-pd-1 or PD-L1 antibodies (194). In addition, PD-L1 expression on malignant myeloma plasma cells was related to an increased risk of MM (195).

However, the role of PD-L1/PD-1 axis in MM is still debated, the clinical outcomes of PD-1/PD-L1 inhibitors alone for MM are not very encouraging, the combination of PD-1/PD-L1 inhibitors with other drugs for multiple myeloma appears to be promising (196, 197). Recent study showed that pembrolizumab (anti-PD-1) in combination with belapectin (Galectin-3 Inhibitor) significantly enhanced the activation of effector memory T cells and the percentage of effector memory T cell proliferation in MM patients. Moreover, pembrolizumab in combination with belapectin was associated with fewer immune-related adverse events compared to pembrolizumab monotherapy (198)). Moreover,PD-1inhibitor in combination with CD38 monoclonal antibody was also a promising strategy for the treatment of CD38-positive MM (194). In fact, *in vitro* experiments have also demonstrated that PD-1/PD-L1 inhibitors directly enhance NK cell- and T cell-mediated immune responses against MM, and lenalidomide (immunomodulator) significantly enhanced such immune responses (199). Overall, PD-1/PD-L1 expressions in MM have shown an important clinical significance and its inhibitors have a certain potential in the treatment of MM, but the conclusions of their effectiveness are inconsistent and more rigorous clinical and basic studies are required to confirm that.

### Lymphoma

Lymphoma is a kind of heterogeneous lymph-like malignancy (200).The World Health Organization classifies lymphomas into more than 80 subtypes in 2017 year based on their morphology, immunophenotype, genetic lesion, molecular profile, clinical features and cell type of origin, such as B cell lymphoma, T cell lymphoma, Hodgkin’s lymphoma and so on (200, 201). Preliminary clinical data suggested that checkpoint inhibitors were a promising therapeutic strategy for certain lymphoid malignancies. However, the expression level and role of PD-1/PD-L1 in lymphoma cells and tumor microenvironment varied depending on the subtype (202). For example, increased infiltration of PD-1+ tumor-infiltrating lymphocytes (TILs) was a positive prognostic predictor in diffuse large B-cell lymphoma (DLBCL) but not in Hodgkin’s lymphoma (HL) (202). The NK cell-associated and monocyte/macrophage-associated immune escape due to the PD-1/PD-L1 pathway was more prominent in HL than DLBCL (203).

Anyway, PD-1/PD-L1 inhibitors have still made some promising achievements in the research and treatment of lymphoma. For instance, a clinical trial suggested that PD-L1 may be the most promising soluble biomarker for classical Hodgkin lymphoma (CHL) (204). The objective response rate(ORR) to nivolumab was 66.3% (53/80) in a multicenter, multicohort, single-arm phase 2 trial for classical Hodgkin’s lymphoma after failure of autologous stem cell transplantation and brentuximab vedotin (65). Moreover, GLS-010, a recombinant human anti-programmed death-1 monoclonal antibody, has demonstrated favorable response and safety in clinical trials for the treatment of advanced solid tumors or lymphomas (77). In a recent phase 2 study, pembrolumab significantly improved the PFS and OS in patients with relapsed/refractory (R/R) CHL after autologous stem cell transplantation (ASCT) and achieved 82% PFS at 18 months and 100% OS at 18 months, which suggested that pembrolumab is a promising approach for post-ASCT consolidation in patients with R/R CHL (205). Furthermore, the results of a small phase 1b study showed that the ORR was 36% in patients with R/R diffuse large B-cell lymphoma (DLBCL) treated with nivolumab (67). However, in a subsequent larger phase 2 study, the ORR to nivolumab treatment was only 10% and 3% respectively, median response time was 11 and 8 months respectively in patients with R/R DLBCL who are ineligible for autologous hematopoietic cell transplantation (AHCT) or experienced failure with AHCT (87). Taken together, considering the diversity and complexity of lymphomas, more precise and individual clinical trials are necessary to elucidate the role of PD-1/PD-L1 inhibitors for the treatment of lymphomas in the future.

### Problems and prospects

Currently, there is an increasing number of studies targeting PD-1/PD-L1 immune checkpoint inhibitors in human cancers including solid tumors and hematological malignancies. PD-1 and PD-L1 are expressed in tumor-infiltrating immune cells and most solid tumors, and they are closely associated with tumor development and prognosis (206–209). PD-L1-positive patients have a significantly lower 5-year survival rate than patients with non-PD-L1-positive tumors, and PD-L1 expression is an independent prognostic indicator (210). PD-L1 expression is associated with many factors, such as age, tumor size, depth of infiltration, lymph node metastasis, lymphovascular infiltration, venous infiltration and disease stage. Recent studies have shown that PD-L1 and PD-1 expressions are often closely linked. In patients with high PD-L1 expression, PD-1 levels in T cells are also high, which may be an intrinsic factor for the immune escape of tumors (210). Activated T cells play a key role in tumor suppression. PD-1 is mainly expressed in activated T cells and inhibits T cell function through binding to PD-L1, thereby promoting immune escape (22).Therefore, blockade of interaction between PD-1 and PD-L1 can significantly enhance immune function and inhibit tumor growth. A multicenter phase 1 trial showed that intravenous anti-PD-L1 antibody significantly inhibited tumor progression (objective remission rate of 6-17%) and prolonged disease stability (12-41% at 24 weeks) (211).

Although PD-1/PD-L1 expression is closely associated with tumor progression and treatment, using PD-1/PD-L1 as the only predictive biomarker for cancer immunotherapy still remains problematic. For example, the low accuracy of PD-L1 detection brings unnecessary obstacles for examination and anti-PD-L1 treatment in patients. The main reasons are considered as follows: Firstly, different studies may use antibodies of varying sensitivity. Secondly, the criteria for positive PD-L1 staining were inconsistent across studies. Thirdly, the expression level of PD-L1 in different sites of tumor tissues is variable, and even if the same sites are sampled at different times, the results of PD-L1 detection can be affected (212). Fortunately, some studies have found that some other factors can work together with PD-L1 as biomarkers to better predict the responsiveness of anti-PD-1/PD-L1 therapy. For example, high expression of TMB (tumor mutational burden), T-cell-inflamed gene-expression profile(GEP) and PD-L1 together reflect the potential for higher response of pembrolizumab in various types of cancers (57).

Taken together, PD-1/PD-L1 immune checkpoint inhibitors have been widely used in the treatment of a variety of cancers (64, 65, 68, 71, 75, 76). However, serious challenges still remain, such as the small number of beneficiary populations, primary and acquired drug resistance, lack of predictive and prognostic biomarkers, and treatment-related adverse effects (22). In addition, there are few predictive biomarkers that can identify the type of patients who will benefit from treatment (213, 214). Moreover, PD-L1 expression is heterogeneous and dynamic in tests with different antibodies and different scoring criteria complicate the interpretation of the test results (215–217). Therefore, considering the multifactorial characteristics of tumor immune crosstalk, prediction models based on comprehensive biomarker determination theory may be more feasible for future applications. Finally, in response to the treatment resistance of PD-1/PD-L1 blockers, some investigators believe that combined therapy, nanoimmunotherapy and intestinal microbial therapy may be promising therapeutic strategy (218). In any case, PD-1/PD-L1 Immune-checkpoint inhibitors definitely have wildly application prospects and clinical value in the treatment of human cancers.

## Author contributions

QT, YC, and XL drafted the manuscript and were involved in data analysis in the whole manuscript. SL, YS, and YY were involved in technical support and revised the manuscript. WW and LH contributed to funding and writing suggestions. SW revised the manuscript and contributed to the conception and design of the work. All authors approve it for publication and agree to be accountable for all aspects of the work.

## Funding

This work was supported by the grants from the Natural Science Foundation of China (81974543, 81903991, 81703551, 81871863), the Natural Science Foundation of Guangdong Province (2019A1515011362, 2021A1515410007, 2021A1515220023), the Guangzhou science and technology plan project (202002030155, 202102010160), the Scientific Research Project in Universities of Guangdong Provincial Department of Education (2020KTSCX029), the Chinese medicine science and technology research project of Guangdong Provincial Hospital of Chinese Medicine (YN2019MJ09, YN2019QJ06), the Guangdong Provincial Key Laboratory of Clinical Research on Traditional Chinese Medicine Syndrome (ZH2020KF03), the Key project of State Key Laboratory of dampness syndrome of Chinese medicine (SZ2021ZZ38, SZ2021ZZ29), the Science and Technology Planning Project of Guangdong Province (2017B030314166) and the Research Fund for Bajian Talents of Guangdong Provincial Hospital of Chinese Medicine(BJ2022KY13).

## Conflict of interest

The authors declare that the research was conducted in the absence of any commercial or financial relationships that could be construed as a potential conflict of interest.

## Publisher’s note

All claims expressed in this article are solely those of the authors and do not necessarily represent those of their affiliated organizations, or those of the publisher, the editors and the reviewers. Any product that may be evaluated in this article, or claim that may be made by its manufacturer, is not guaranteed or endorsed by the publisher.
